# Fatal Toxic Megacolon Following Nivolumab and Ipilimumab Therapy in Metastatic Melanoma: A Case Report

**DOI:** 10.7759/cureus.86902

**Published:** 2025-06-28

**Authors:** Hibiki Fujiwara, Takuya Maeda, Takehiko Katsurada, Hideyuki Ujiie

**Affiliations:** 1 Department of Dermatology, Hokkaido University, Sapporo, JPN; 2 Department of Gastroenterology, Hokkaido University, Sapporo, JPN

**Keywords:** immune checkpoint inhibitor colitis, immune-checkpoint inhibitors, immune-related adverse event, melanoma treatment, toxic megacolon

## Abstract

In this case report, we describe a 76-year-old man with advanced malignant melanoma who developed severe colitis and toxic megacolon following nivolumab plus ipilimumab therapy. Despite treatment with prednisolone, infliximab, and vedolizumab, the patient’s condition deteriorated, resulting in hypoalbuminemia and unfeasibility of surgical intervention. Elevated C-reactive protein (CRP) levels served as a critical diagnostic clue, prompting abdominal radiography, which subsequently confirmed the diagnosis of toxic megacolon. This case highlights a rare but life-threatening complication of immune checkpoint inhibitors and underscores the challenges in managing refractory immune-related colitis. Given the increasing use of immune checkpoint inhibitors in clinical practice, this report will be of significant interest to readers by emphasizing the importance of timely recognition, CRP monitoring, and intervention to prevent fatal outcomes in similar cases.

## Introduction

Immune checkpoint inhibitors (ICIs), including cytotoxic T-lymphocyte-associated antigen 4 (CTLA-4) and programmed death 1 (PD-1) inhibitors, have revolutionized the treatment of various malignancies by enhancing T cell-mediated antitumor responses. While these agents have shown remarkable clinical benefits, they are also associated with a spectrum of immune-related adverse events (irAEs) resulting from nonspecific immune activation. Among these, gastrointestinal irAEs are relatively common, with colitis being one of the most frequently observed toxicities, especially in patients receiving combination therapy with nivolumab (anti-PD-1) and ipilimumab (anti-CTLA-4). The incidence of colitis associated with dual ICI therapy is notably higher than with monotherapy and ranges from 5% to 8% for grade 1-3 events, although grade 4 events remain rare [1]. Furthermore, there are reports indicating that fatal cases of irAE colitis account for only 0.13% of all cases [2].

ICI-induced colitis can range in severity from mild diarrhea to fulminant colitis requiring hospitalization. While most cases respond to immunosuppressive therapy such as corticosteroids, a subset of patients develops steroid-refractory colitis, necessitating additional treatments such as infliximab or vedolizumab. In rare instances, severe ICI-induced colitis may progress to toxic megacolon [3], a life-threatening condition characterized by colonic dilatation, systemic toxicity, and risk of perforation. Toxic megacolon typically requires surgical intervention, but poor performance status or comorbidities may preclude operative management.

Reports of toxic megacolon in the context of ICI-induced colitis are extremely scarce [3], and fatal outcomes remain largely undocumented in the literature. Here, we present a rare and fatal case of toxic megacolon that developed after combined nivolumab and ipilimumab therapy for metastatic melanoma. Despite escalation from corticosteroids to biologic therapies, including infliximab and vedolizumab, the patient’s condition deteriorated. This case underscores the importance of early recognition of high-risk features, timely escalation of care, and multidisciplinary coordination in managing severe irAE colitis.

## Case presentation

**Figure 1:**
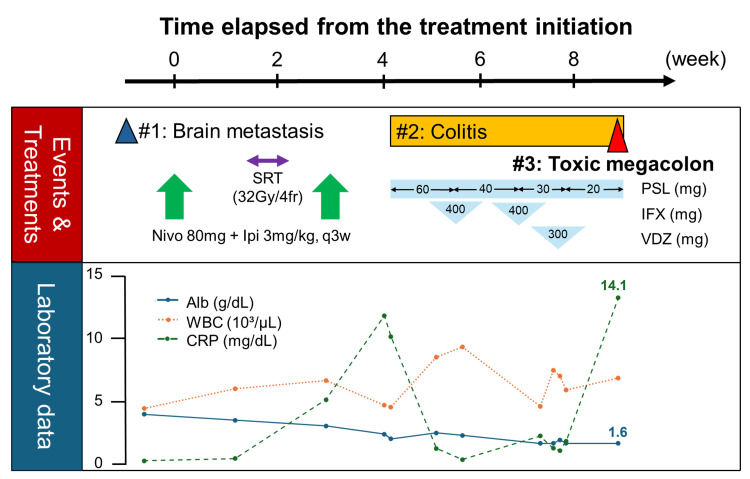
Schematics of the clinical course. SRT, stereotactic radiation therapy; Nivo, nivolumab; Ipi, ipilimumab; PSL, prednisolone; IFX, infliximab; VDZ, vedolizumab; Alb, albumin; WBC, white blood cell; CRP, C-reactive protein.

**Figure 2:**
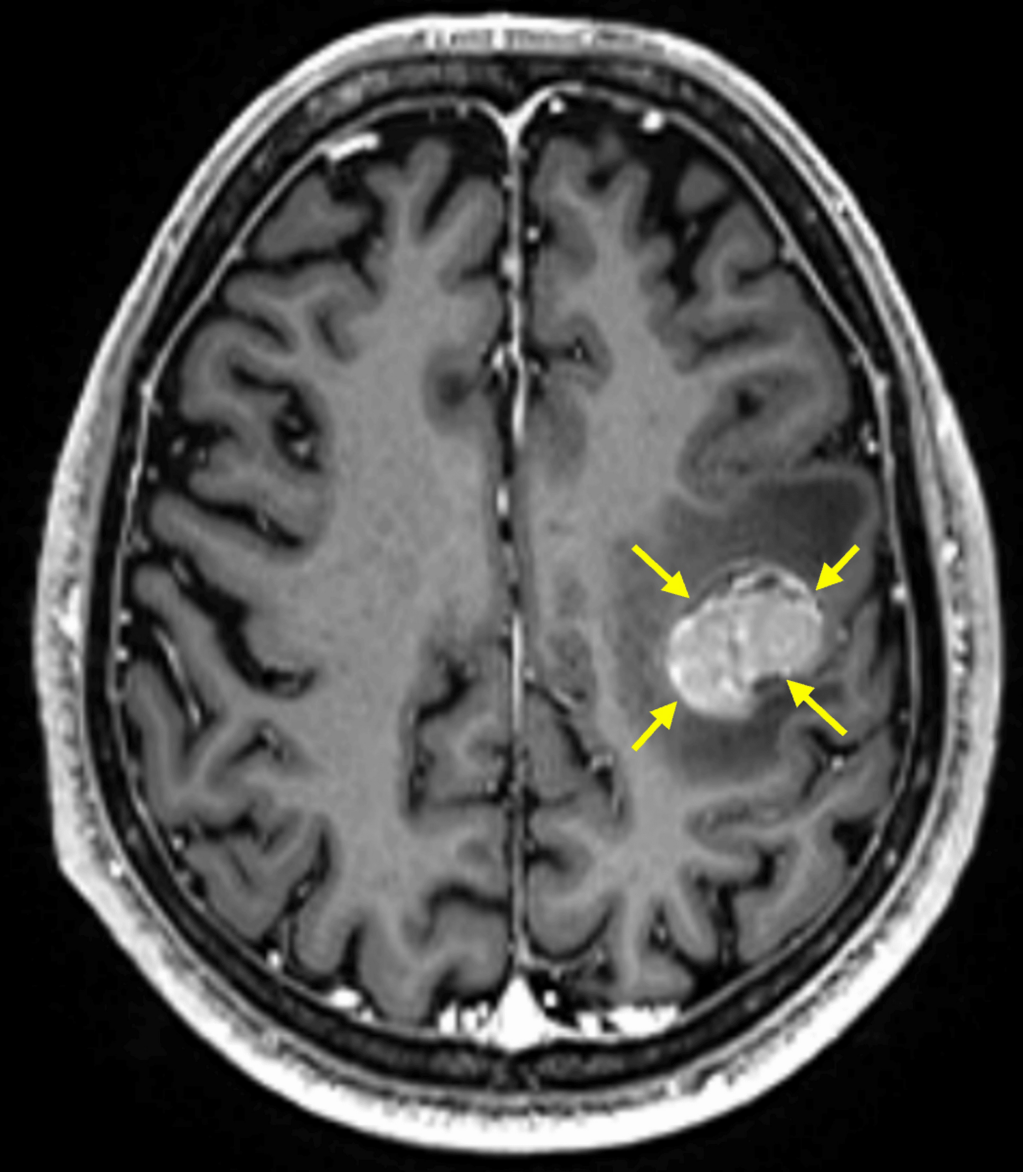
Image of the brain metastasis A T1-weighted image of gadolinium-enhanced brain MRI shows a brain metastasis (contrast-enhancing mass; idicated by yellow arrows) in the left frontal lobe with surrounding edema (low-signal area).

A 76-year-old Japanese man was diagnosed with malignant melanoma on the right sole. Based on local excision and sentinel lymph node biopsy, the tumor was diagnosed as pT4bN3aM0, stage IIID melanoma. He received postoperative adjuvant therapy with dabrafenib and trametinib. However, symptomatic brain metastases were detected immediately after the completion of this therapy (Figures 1, 2).

**Figure 3:**
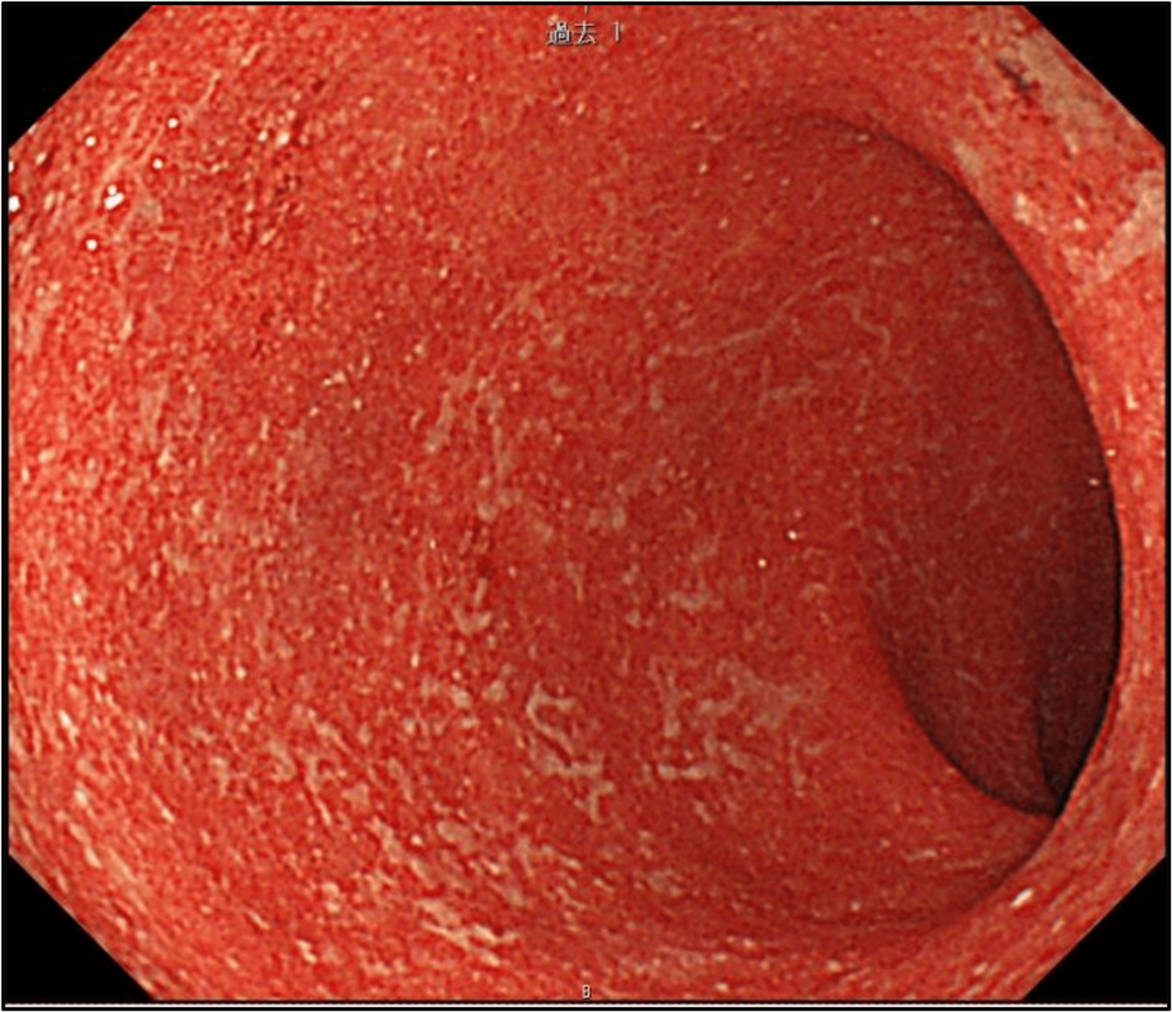
Colonoscopy images Colonoscopy images show diffuse erosion and the loss of the vascular pattern. Due to limitations in endoscopic imaging, this figure shows the maximum extent of the lesion that could be captured.

**Figure 4:**
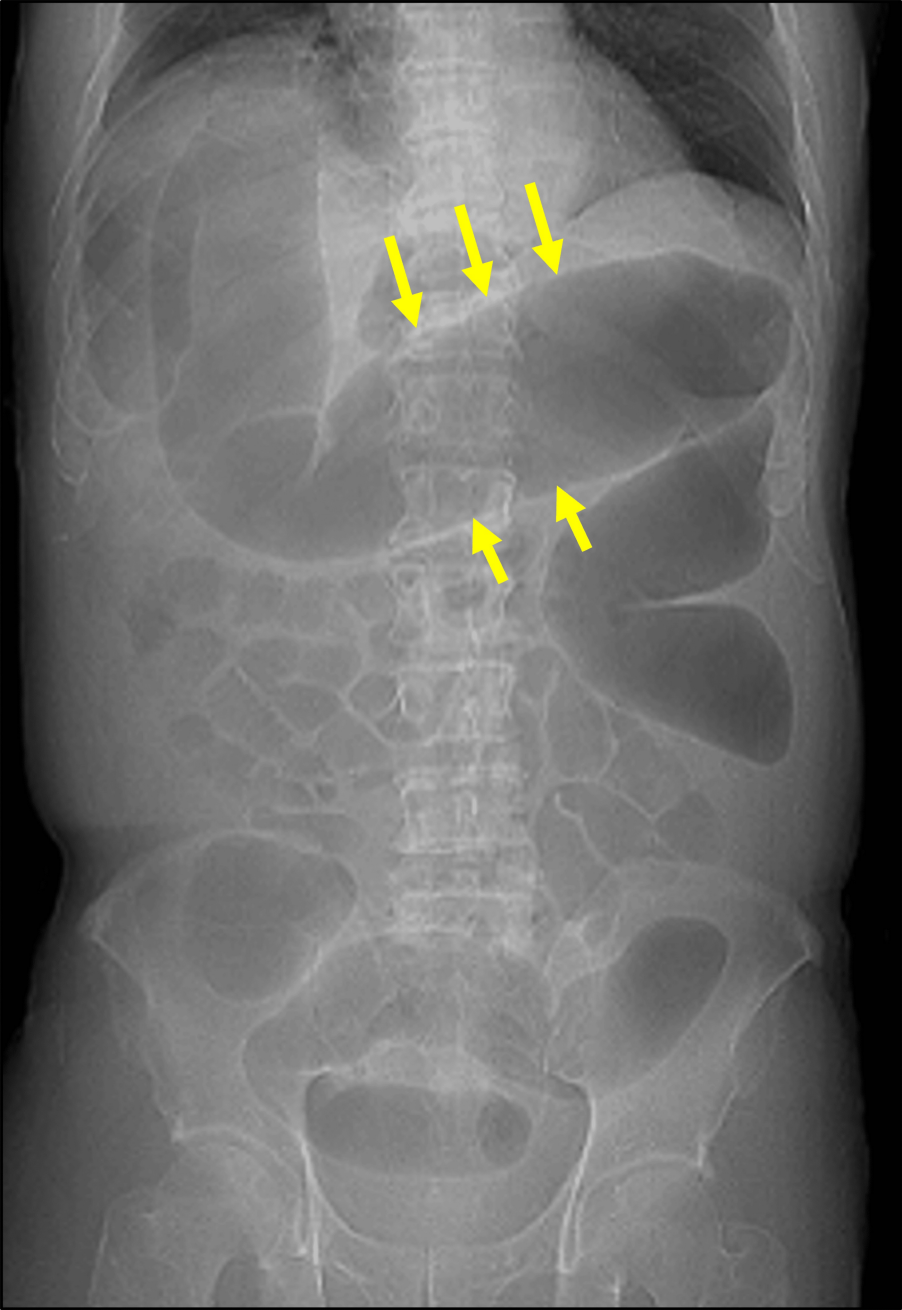
An abdominal radiography An abdominal radiography shows marked colonic dilatation and the loss of haustra (#3, yellow arrows), leading to the diagnosis of toxic megacolon.

Nivolumab plus ipilimumab was then initiated (Day 1). Figure 1 summarizes the clinical course. The first cycle of treatment was completed without complications; however, bloody diarrhea developed immediately after the second cycle (Day 31). A colonoscopy confirmed the diagnosis of irAE colitis (Figure 3), prompting the initiation of prednisolone therapy. Although initial treatment resulted in a reduction in C-reactive protein (CRP) levels, bloody diarrhea persisted and hypoalbuminemia progressed (reference range: 3.8-5.3 g/dL). Infliximab and vedolizumab were subsequently administered; however, the symptoms did not improve. As CRP levels rose again, an abdominal radiograph was performed, confirming the diagnosis of toxic megacolon (Day 66, Figure 4). A detailed timeline and laboratory findings are summarized in Table 1.

**Table 1:** Clinical timeline and laboratory trends Alb, albumin; CRP, C-reactive protein; Nivo, nivolumab; Ipi, ipilimumab; irAE, immune-related adverse event; G, grade; PSL, prednisolone; IFX, infliximab; US, ultrasonography; VDZ, vedolizumab; CT, computed tomography.

DAY	Clinical Events and Interventions	Alb (g/dL)	CRP (mg/dL)
-7	-	4.1	0.11
1	Nivo 80mg + Ipi 3mg/kg ①	-	-
22	Nivo 80mg + Ipi 3mg/kg ②	3.1	5.36
31	Bloody diarrhea, Colonoscopy: irAE colitis G3 (Fig 3) PSL 60mg/day, fasting	2.4	12.57
35	Persistent bloody diarrhea	2.3	2.59
37	Colonoscopy: colitis progression	-	-
38	IFX 400mg ①	2.5	1.16
39	PSL 40mg/day	-	-
42	Resumption of oral intake	2.3	0.22
43	US: unresolved	-	-
45	-	2.2	0.10
49	-	2.0	0.46
51	IFX 400mg ②, PSL 30mg/day	-	-
53	Bloody diarrhea, fasting	1.6	2.24
55	Colonoscopy: colitis persistent	-	-
56	VDZ 300mg ①	1.6	1.19
57	Resumption of oral intake	1.9	1.00
58	PSL 20mg/day	1.6	1.80
62	-	1.6	3.76
64	-	1.8	6.09
66	CT: Toxic megacolon (Fig 4)	1.6	14.11
67-	Palliative care initiated		
112	Died due to intestinal bleeding	-	-

Surgical intervention was deemed unfeasible due to the patient’s poor general condition and hypoalbuminemia, and prednisolone treatment was continued; however, the patient died due to intestinal bleeding at week 16.

## Discussion

ICIs exert their antitumor effects by enhancing T cell activation. Specifically, they promote the expansion of effector T cells and suppress the function of regulatory T cells, thereby generating a pro-inflammatory immune environment. This immune dysregulation is considered a key mechanism in the development of ICI-induced colitis [4]. In affected patients, histological examination of colonic tissue often reveals dense infiltration of activated CD4⁺ and CD8⁺ T cells. These infiltrates are typically accompanied by increased levels of proinflammatory cytokines, including IL-6/17, interferon-gamma (IFN-γ), and tumor necrosis factor alpha (TNF-α), which are thought to mediate mucosal injury and contribute to the severity of colitis [4,5]. In patients with severe colitis, dysregulated neuromuscular signaling - partly driven by increased nitric oxide production in inflamed mucosa - can impair colonic smooth muscle contraction. This functional disruption may lead to reduced peristalsis and intraluminal stasis of gas and fecal material, thereby facilitating the progression to toxic megacolon [6].

Prednisolone is the first-line treatment for irAE colitis, with infliximab and vedolizumab (sometimes taking slower effect) considered as additional options if the initial treatment is ineffective [7]. Despite these treatments, the colitis did not improve in our case. One possible explanation is that by the time infliximab was initiated, mucosal injury and immunological dysregulation had already progressed beyond the point where TNF-α blockade could reverse the inflammatory cascade. Similarly, vedolizumab did not result in clinical improvement, which may reflect a distinct mechanism of resistance. As an α4β7 integrin blocker, vedolizumab prevents lymphocyte trafficking to the gut but has limited impact on already-resident effector T cells. In this case, colitis may have been driven predominantly by locally activated CD8⁺ and CD4⁺ T cells and cytokine-mediated injury, rather than ongoing recruitment of new immune cells. Furthermore, the extensive epithelial damage and malnutrition may have further compromised mucosal healing, narrowing the therapeutic window for vedolizumab efficacy. Clinical guidelines recommend CT imaging when toxic megacolon is suspected. In this case, the decision to proceed with a CT scan was precipitated by an elevation in CRP levels. Prolonged irAE colitis can lead to hypoalbuminemia, potentially making surgical intervention unfeasible. Therefore, when disease progression - such as toxic megacolon - is suspected, early and proactive imaging studies such as CT should be strongly considered.

There is a prior report of a 53-year-old man who developed grade 3 irAE colitis and toxic megacolon two months after initiating nivolumab plus ipilimumab. He responded well to two doses of infliximab and recovered without the need for surgery [3]. In contrast, our 76-year-old patient developed bloody diarrhea immediately after the second ICI cycle. Despite treatment with both infliximab and vedolizumab, his condition did not improve. Persistent CRP elevation, progressive hypoalbuminemia, and worsening general condition ultimately rendered surgical treatment unfeasible, and he succumbed to intestinal hemorrhage. Differences in age, timing of onset, treatment responsiveness, and nutritional status may have contributed to the divergent clinical courses. This report has several limitations. As neither histopathological examination nor autopsy was performed in this case, the diagnosis was made solely on clinical grounds. Furthermore, since this is a single case report, general conclusions cannot be drawn without collecting and performing an integrated analysis of multiple similar cases.

## Conclusions

In conclusion, we described the clinical course of toxic megacolon associated with nivolumab plus ipilimumab therapy. Elevated CRP during treatment provided a diagnostic clue. Four weeks of persistent treatment-resistant irAE colitis led to toxic megacolon and a loss of albumin, which proved fatal for this patient. These findings underscore the importance of early recognition and aggressive management of irAE colitis. Serum albumin levels and general condition may serve as prognostic indicators in determining the feasibility of surgical intervention. When toxic megacolon is diagnosed, a multidisciplinary approach - including early surgical consultation - should be considered before the patient’s condition deteriorates beyond recovery.
